# Surviving antibiotic treatment as a gut bacterium: genomic characterization of an *Enterobacter cloacae*

**DOI:** 10.1186/s12863-025-01346-x

**Published:** 2025-08-12

**Authors:** Anna Baborski, Stefanie A. Barth, Elke Martina Jung, Frank Bloos, Jürgen Rödel, Bettina Löffler, Michael Bauer, Anne Busch

**Affiliations:** 1 Department of Anaesthesiology and Intensive Care Medicine, University Hospital Jena, Am Klinikum 1, 07747 Jena, Germany; 2 Cluster of Excellence Balance of the Microverse, Friedrich Schiller University Jena, Jena, Germany; 3 Institute of Molecular Pathogenesis, Friedrich-Loeffler-Institut/Federal Research Institute for Animal Health, Naumburger Str. 96a, 07743 Jena, Germany; 4 Department of Medical Microbiology, University Hospital Jena, Am Klinikum 1, 07747 Jena, Germany; 5 Institute of Microbiology, Friedrich Schiller University Jena, Jena, Germany; 6 Theoretical Microbial Ecology, Friedrich Schiller University, Jena, Germany

**Keywords:** *Enterobacter cloacae*, Sepsis, Biofilm, Antibiotic resistance

## Abstract

*Enterobacter cloacae* complex is a group of common opportunistic pathogens on intensive care units. On intensive care units sepsis is treated with high doses of antibiotics. This treatment does not only eliminate pathogenic bacteria but parts of the microbiome community as well. This leads to an imbalance of the gut microbiome. However, some bacteria can survive such treatment due to certain survival and resistance mechanisms. Not only antibiotic resistance mechanisms but also forming strong communities via biofilm formation promotes cell survival. Here, we investigated the properties of the isolate AT70PIP076 from a sepsis patient treated with piperacillin and tazobactam. After biochemical analysis and MALDI-TOF analysis, the strain was found to be *Enterobacter cloacae.* In addition to in vitro, antimicrobial susceptibility testing the genome was further investigated in situ regarding antibiotic resistance. Further live/dead staining was performed, and the biofilm formation was investigated using confocal laser microscopy (cLSM). The genome shows the presence of biofilm-associated genes *EU554560*, *bcsABZC_AP010953*, *ehaB*, *KF662843*, and *crl*. The understanding of the underlying mechanism of survival of potential pathogens might contribute to elucidate potential treatment options.

**Objectives**Genomic analysis of a bacterium that can survive antibiotic treatment within the gut of an antibiotictreated patient to elucidate survival and resistance mechanisms.

**Data description**The isolate AT70PIP076 was isolated in 2021 from feces collected from a patient treated with Piperacillin and tazobactam. Whole genome DNA was isolated using the Nextera DNA Flex microbial colony extraction protocol and the Nextera Flex DNA preparation kit according to the manufacturer’s instructions. Following paired-end sequencing was performed on the MiSeq platform (Illumina, Inc., San Diego, CA, USA) using a 300-cycle MiSeq reagent kit and a read length of 151 bp. Contamination check and identification of 16 S RNA sequences was done by using ContESt16S. The genomic sequence contained 4,988,237 bp and the G + C content is represented at 54.80%. This genome and its associated data set will serve as a useful resource for further analyses.

## Objective

The gut microbiome is a commensal community that fulfills several functions in the human body including immune response, vitamin synthesis, and digestion [1, 2]. After sepsis which is treated with high doses of antibiotics, the gut microbiome falls under disbalance. As sequelae of sepsis, *Enterobacteriaceae* are sometimes found in the bloodstream as secondary infection. These are suspected to derive from the surviving microbiome community within the patient’s gut [3]. Bacteria that can survive high doses of antibiotics as used in sepsis treatment need to have survival mechanisms. In addition to direct antibiotic resistance mechanisms biofilm formation was found to promote the survival rate of gut bacteria under antibiotic treatment.

The bacterial isolate, AT070PIP076, was collected from human feces from a sepsis patient treated with piperacillin and tazobactam at the University Hospital Jena, Germany, in 2021. This isolate raised special interest due to unclear identification at first sight. MALDI-TOF identified the species as *Enterobacter cloacae* whereas genomic identification using SpeciesFinder presents the species as *Kosakonia sp*. The objective of this study is to report the draft genome sequence of a bacterium that survives in the gut of a sepsis patient treated with high doses of antibiotics. Moreover, further underlying survival mechanism besides antibiotic resistance have been analyzed.

**Table 1:** Overview of data files/data sets

Label	Name of data file/data set	File types (file extension)		Data repository and identifier (DOI or accession number).
Data file 1	AT070PIP076 nucleotide record	Fasta file (.fasta)		NCBI https://identifiers.org/ncbi/insdc:JAVGVQ000000000 [4]
Data file 2	AT070PIP076 SRA record, raw Illumina MiSeq reads	Fastq file (fastq.gz)	NCBI https://identifiers.org/ncbi/insdc.sra:SRR31899943 [5]	

Isolated bacteria were cultured and grown on Columbia blood agar with 5% sheep blood (Beckton, Dickinson GmbH) at 37 °C under aerobic and anaerobic conditions indicating facultative anaerobic characteristics of AT070PIP076.

Whole genome DNA was isolated using the Nextera DNA Flex microbial colony extraction protocol and the Nextera Flex DNA preparation kit according to the manufacturer’s instructions. Following paired-end sequencing was performed on the MiSeq platform (Illumina, Inc., San Diego, CA, USA) using a 300-cycle MiSeq reagent kit and a read length of 151 bp. Contamination check and identification of 16 S RNA sequences was done by using ContESt16S. The genomic sequence contained 4,988,237 bp and the G + C content is represented at 54.80%. Species identification based on genome analysis using SpeciesFinder [6] was detected as *Kosakonia sp.* Since MALDI-TOF detected this isolate as *Enterobacter cloacae* we ran further biochemical investigations.

Biochemical analysis of the isolate AT070PIP076 was performed using API^®^ 20E biochemical analysis kit.

(bioMérieux, Marcy-l’Étoile, France). The strain has a positive reaction for β-galactosidase, arginine dihydrolase, ornithine decarboxylase, citrate utilization, and acetoin production but it is negative for lysine decarboxylase, H_2_S production, urease, tryptophane deaminase, indole production, and gelatinase. It can ferment or oxidate glucose, mannitol, sorbitol, rhamnose, saccharose, melibiose, amygdalin, and arabinose but not inositol. A positive result is also shown for catalase and cytochrome oxidase activity. Due to the biochemical properties of the bacterium, it can be classified as *Enterobacter cloacae*.

The antimicrobial susceptibility test was performed using the Vitek Compact system (bioMérieux, Marcyl’Étoile, France). Susceptibility and resistance were determined by clinical MICs according to EUCAST breakpoints. Antimicrobial susceptibility was assessed by the EUCAST breakpoints by determining the clinical MICs (Minimum inhibitory concentration) using the Vitek Compact system (bioMérieux, Marcyl’Étoile, France). The strain was categorized as resistant to piperacillin (MIC ≤ 4 mg/L) resistant to piperacillin + tazobactam (MIC ≥ 4 mg/L), resistant to ampicillin (MIC of 18 mg/L), resistant to ampicillin + sulbactam (MIC ≤ 2 mg/L), resistant to cefpodoxime (MIC ≤ 1 mg/L), resistant to cefotaxime (MIC ≤ 1 mg/L), resistant to ceftazidime (MIC ≤ 1 mg/L) and resistant to tigecycline (MIC ≤ 0.5 mg/L). On the other hand, the strain is susceptible to gentamicin (MIC ≤ 1 mg/L), susceptible to imipenem (MIC ≤ 0.25 mg/L), susceptible ertapenem (MIC ≤ 0.5 mg/L), susceptible to ciprofloxacin (MIC ≤ 0.25 mg/L) and susceptible to meropenem (MIC ≤ 0.25 mg/L) [7]. Most interestingly this survivor of antibiotic treatments belongs not to the group of carbapenemase-producing *Enterobacter* spp. that are known for nosocomial infections.

The genome was mapped against a collection of biofilm-associated genes [8] using Geneious (Geneious Prime 2021, https://www.geneious.com). The genome shows the presence genes related to biofilm formation *EU554560*, *bcsABZC_AP010953*, *ehaB*, *KF662843* and *crl*.

## Limitations

This data note is limited to a single isolate which was specifically analyzed. Currently, further investigations are done in the same study regarding these results. However, further investigations in larger sample collections are required. The length of the 16 S rDNA of 536 bp might influenced genomic species identification.

## Data Availability

https://www.ncbi.nlm.nih.gov/bioproject/?term=PRJNA1009013/, https://www.ncbi.nlm.nih.gov/biosample/SAMN37141839/, https://www.ncbi.nlm.nih.gov/datasets/genome/GCA_030971765.1/.

## Abbreviations

Bp: base pairs
cLSM: confocal laser scanning microscopy
EUCAST: European Committee on Antimicrobial Susceptibility Testing
MALDI-TOF: Matrix-assisted Laser Desorption/Ionization, MALDI; time of flight, TOF
MIC: Minimum inhibitory concentration
